# Flame-Retardant-Functionalized Pineapple Leaf Fibers for Sustainable Acoustic Absorption

**DOI:** 10.1021/acsomega.6c02520

**Published:** 2026-06-24

**Authors:** Sunisa Suwatthi, Kritsana Janyajaraskul, Jitlada Boonlertsamut, Nakarin Subjalearndee, Varol Intasanta, Chutima Vanichvattanadecha

**Affiliations:** † Advanced Composite and Nanotextiles Research Team, National Nanotechnology Center, 61191 National Science and Technology Development Agency, Pathum Thani 12120, Thailand; ‡ Chemistry Department, Faculty of Science, 133942 Chulalongkorn University, Bangkok 10330, Thailand

## Abstract

Pineapple leaf fibers (PF), a byproduct of pineapple cultivation, inherit high cellulosic content and porosity that offer a sustainable and cost-effective alternative for manufacturing nonwoven materials with excellent acoustic absorption properties. However, one major drawback of natural-based acoustic materials is their high flammability due to their high cellulosic content. This presents a challenge in complying with fire safety standards required in the building industries. In this work, extractions of PF via an enzymatic and a combined enzyme–chemical treatments were performed before finishing the extracted PF with phosphorus-based flame-retardant agents (FR) and an antistatic oil (oil) additive-based finishing agents using the simple padding process. The finished fibers are physically and chemically characterized by mechanical testing, Fourier-transform infrared spectroscopy (FTIR), scanning electron microscopy, and thermogravimetric analysis. The flame-retardant properties were evaluated by the modified method aligned with vertical UL 94 standard testing, while the acoustic absorption performance was measured using ASTM E1050–95 standard. The results showed that PF treated with oil prior to FR in both staple fibers and nonwoven samples demonstrated superior flame-retardant performance, which was attributed to the increased surface energy examined by contact angle measurements after oil treatment, leading to improved dispersion and physical attachment of the FR. In addition, noise reduction coefficient values of the flame-retarded PF samples were found to be in the range from 0.55 to 0.60, which indicated that the functional finishing did not deteriorate the acoustic performance. Furthermore, the PF needle-punched nonwoven exhibited superior performance in terms of flame retardancy, acoustic absorption, and compression resilience properties. Based on overall functionality, morphological stability, and cost considerations, the flame-retarded PF samples show strong potential as a sustainable flame-retarded sound absorbing materials.

## Graphical abstract

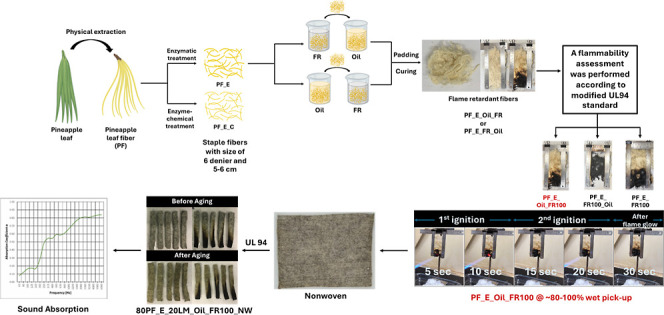

## 1 Introduction

Porous synthetic materials such as mineral wool, polyurethane (PU), and polyester serve as key raw materials for sound-absorbing panels which are widely used in the building industries.^1–3^ In 2005, glass fiber and mineral wool dominated the insulation material market in Europe, accounting for 60% of the market, while organic foamy materials such as polystyrene (PS) and PU accounted for 27%.^4^ In recent years, the European insulation market experienced notable shifts. Expanded PS had emerged as a leading insulation material, capturing 41% of the market, followed by extruded PS with a 38.5% market share. On the other hand, mineral wool and PU market share decreased to approximately 14% and 6.5%, respectively.^5^ Despite the decline in market share, glass fiber and mineral wool have significant advantages in acoustic and thermal insulation performance. However, it cannot be ignored that some potential human health problems occur due to skin irritation and deposition in the lung alveoli caused by inhaling these fibers and particles.^6^ In addition, conventional synthetic materials have significant environmental impacts than natural fibers.^7^ For instance, synthetic fiber is usually made from high-temperature manufacturing processes, and the source of synthetic fiber is often taken from petrochemical sources, thus producing a significant amount of carbon footprints.^2,8^ Hence, it is meaningful to seek environmentally friendly materials to substitute conventional sound absorption materials. Importantly, promoting the use of natural fibers in acoustic applications also aligns with broader efforts to reduce plastic dependency and mitigate plastic-related environmental pollution. Moreover, continuous innovation and increasing emphasis on eco-friendly materials are expected to drive the market for sound-absorbing materials toward sustainability by 2035.^9^

Natural fiber-based products can be considered as ideal acoustic products because of their environmentally benign, lightweight, and highly efficient sound absorption capability^7,10^ Furthermore, the number of research studies focusing on natural fiber-based acoustic materials, including pineapple leaf fiber (PF), has increased significantly since 2014, reflecting the growing interest in sustainable and high-performance sound-absorbing materials.^11^

Several studies have been conducted to evaluate the acoustic performance of natural fiber-based sound absorbers, particularly focusing on their absorption characteristics and predictive modeling approaches.^12,13^ Natural fiber composites have demonstrated promising sound absorption capabilities, with experimental results indicating absorption coefficients (the noise reduction coefficient (NRC)) value of 0.6 or higher.^14–16^ PF is gaining attention in various industries such as textile, construction, furniture, and paper industries due to their strong mechanical properties,^17–19^ high cellulose content,^20^ thermal insulation,^11^ and porosity.^21^ In addition, utilizing PF offers an environmentally friendly solution by reducing biomass disposal. Although limited, some research have explored the acoustic potential of PF, reporting favorable sound absorption performance.^22,23^ Previous studies have reported that PF nonwoven with a composition ratio of 30/60/10 (PF/recycled polyethylene terephthalate (r-PET)/low-melt PET) exhibited NRC value of 0.60. When evaluated under equivalent density and thickness conditions, the pineapple fiber (PF) composite demonstrated superior acoustic absorption performance compared with conventional glass fiber insulation, which exhibited a NRC value of 0.57, while also achieving sound absorption characteristics comparable to those of asbestos fiber insulation materials with an NRC value of 0.60.^24^

However, one of the primary limitations of natural-fiber-based acoustic materials is their inherently poor flame resistance due to their high cellulose content. This poses a challenge for meeting fire safety regulations in the building industries. Therefore, integrating effective and environmentally friendly flame-retardant treatments is essential to enhancing the fire performance of these materials while maintaining their acoustic efficiency and sustainability.

Flame-retardant materials play a crucial role in enhancing the fire resistance of polymer materials, particularly textiles, by delaying ignition, promoting char formation, and reducing flame propagation.^25^ For environmental concerns, halogen-free flame retardation has attracted great attention in recent years because halogen containing flame-retardant materials, such as organohalogen compounds, i.e., organochlorines and organobromines, can produce significant smoke and emit toxic gaseous species during combustion.^25,26^ Recent studies have shown that ammonium polyphosphate (APP), a phosphorus-based compound, has gained attention due to its low toxicity and smoke production compared to other substances.^27–29^ APP can significantly enhance the fire resistance property of cellulose-rich fibers under heat exposure by creating a protective barrier that restricts oxygen and heat transfer.^30–32^

The flammability behavior of natural cellulosic fibers is influenced by their chemical composition and structural constituents. In particular, the lignin content plays a significant role in enhancing flame resistance due to its ability to promote the formation of a thermally stable conjugated char layer during combustion.^33^ Nevertheless, lignin is removed or substantially reduced during chemical treatment processes applied to natural fibers, thereby diminishing their inherent fire-resistant properties. To overcome this limitation, numerous studies have focused on improving the flame retardancy of natural fiber through the incorporation of halogen-free flame-retardant systems. For instance, kapok fiber–reinforced polycaprolactone composites have been modified using flame-retardant formulations based on magnesium hydroxide synergistically combined with zinc borate, APP, and antimony trioxide for sound-absorbing applications. The incorporation of these flame-retardant systems significantly enhanced the fire performance of the composites, resulting in limiting oxygen index (LOI) values ranging from 30 to 31.5%.^34^

Similarly, the incorporation of phosphate-based flame retardants has been reported to enhance the flame-retardant performance of pineapple fiber (PF)-reinforced acrylonitrile butadiene styrene composites. Nevertheless, the use of 9,10-dihydro-9-oxa-10-phosphaphenanthrene-10-oxide led to only a modest increase in the LOI.^33^ In another study, PF resin composites containing hybrid mineral flame retardants, namely, magnesium hydroxide and aluminum hydroxide combined with APP, exhibited improved fire resistance in horizontal burning tests, although they failed to comply with the vertical burning test requirements.^35^ These results suggest that further improvement in the flame-retardant properties of PF composites remains a significant challenge.

Surface finishing agents, commonly referred to as spin finishes, which are lubricants, emulsifiers, and antistatic agents, are widely used in the man-made spun fiber and needle punching productions in order to facilitate a reliable and efficient manufacturing process. In previous studies, some studies have investigated the effect of finishing contents related to their physical properties of spun fibers and nonwovens.^35,36^ However, limited research has been conducted on its role as a binder for the finishing agents.

In this study, PF was engineered to exhibit flame-retardant functionality using a phosphorus-based flame-retardant agent (FR) for application as a sound-absorbing material. PF was selected as a sustainable raw material derived from postharvest agricultural waste, emphasizing both environmental and resource-efficiency considerations. Initially, the fibers were extracted using two distinct approaches, a sole enzymatic and a combined enzyme–chemical treatments, to purify fibers and facilitate efficient separation. Following extraction, the fibers were subjected to a finishing process incorporating a phosphorus-based flame retardant, which primarily acts through condensed-phase char formation, together with an antistatic oil that facilitates coating uniformity and interaction with the fiber surface. During the finishing process, the flame-retardant concentration was systematically optimized to achieve effective flame retardancy while preserving the inherently high porosity of the PF structure, which is essential for sound absorption. The resulting functionalized PF materials were subsequently evaluated in terms of both flame-retardant performance and acoustic absorption behavior, allowing correlations to be established between processing conditions, fire resistance, and sound-absorbing efficiency. Moreover, the pineapple nonwoven was fabricated at pilot-scale production with the optimized flame-retardant finishing conditions, and its fire resistance and sound-absorbing performances were also examined.

## 2 Materials and Methods

### 2.1 Materials

Physically extracted PFs were purchased from Chonburi province (Smooth cayenne varieties), Thailand. Xylanase enzyme was purchased from Reach Biotechnology, Pathumthani, Thailand. Sodium hydroxide pellets (NaOH pellets, analytical grade, purity ∼ 98.7%) were purchased from Fisher Chemical. Antistatic oil (oil, amphoteric hydrocarbon, specific gravity = 0.98, pH = 6–7), additive-based finishing agents were kindly supported by Carpets International Thailand Public Company Limited, Thailand. NICCA FI-NONE (flame-retardant materials, FR, nitrogen–phosphorus compound, pH = 5.5) was purchased from Stc Nicca Co., Ltd., Bangkok, Thailand.

### 2.2 Extraction of PFs via Enzymatic and Enzymatic-Chemical Treatments

PF (1 kg) was purified through two different treatment processes. In the enzymatic treatment process, the fibers were treated with xylanase enzyme at a concentration of 2% w/v at a liquor ratio (L/R) of 1:20 for 1 h before incubation at 55 °C for 2 h in a hot air oven.^37^ After the drying process, the fiber was rinsed with water until a neutral pH was achieved before air-drying in a shaded area to prevent direct exposure of the fibers to sunlight. The enzyme-treated PF was labeled as PF_E. In the combined enzymatic-chemical treatment, the fiber was initially treated with 2% w/v xylanase enzyme under the same L/R ratio (1:20) at 55 °C for 1 h, followed by rinsing with water to achieve neutral pH. Subsequently, the fiber underwent chemical treatment with 2% NaOH solution, using the same L/R ratio and temperature for another 1 h. The fiber was then thoroughly rinsed with water to remove residual chemicals and air-dried in a shaded area. The enzyme-chemical treated PF was labeled as PF_E_C.

### 2.3 Flame-Retardant Finishing on Extracted PF

Functionalization of the treated PF was proceeded via two different finishing routes. In the first method, the treated fiber was initially immersed with an antistatic-based finishing (oil) solution of concentration of 100 g/L, an optimal level commonly employed in commercial nonwoven production for 30 s and was subsequently padded at 0.5 bar to remove excess solution by using a padding machine (P-BO Padding Mangle, Copower Technology Co., Ltd.). The fiber was then immersed with a flame-retardant (FR) agent solution at a concentration of 100, 200, or 300 g/L for 30 s before padding at 0.5 bar. After removing the excess solution, the finished fiber was cured at 120 °C for 20 min in a stenter machine (stenter machine lab, MingScape International Co., Ltd.). On the other hand, another finishing method was initially performed by treating the fiber with the FR agent at the different concentration before immersing in the oil solution at 100 g/L, followed by curing at 120 °C for 20 min in a stenter. After the finishing process, the aging step was performed at 70 °C for 168 h to simulate long-term thermal exposure and to assess possible volatilization, migration, or degradation of flame-retardant components. The flame-retardant performances of samples before and after aging were evaluated by the UL 94 method.

### 2.4 Preparation of Nonwoven Samples

For the fabrication of nonwoven sheets intended for sound absorption in technical textile applications, treated pineapple fibers (PF_E) obtained through a biorefining process were first converted into staple fibers. These fibers were then used as the primary raw material to produce nonwoven fabrics by blending pineapple staple fibers with low-melt polyester fibers at a ratio of 80:20 (staple fiber/low-melt polyester). The nonwoven mats were formed using the needle-punching technique, a mechanical bonding process that entangles fibers without the need for chemical binders or heat. This method produces a porous structure suitable for sound absorption and further development of technical textile applications. The resulting nonwoven samples had a basis weight of 1500 GSM.

### 2.5 Flame-Retardant Finishing on Pineapple Nonwoven

The nonwoven sheets were cut into A4-sized specimens and subsequently finished with flame-retardant treatments with optimized condition from staple fiber finishing. The samples were first coated with an antistatic-based finishing agent (oil) at a concentration of 100 g/L for 30 s, followed by padding through a padding machine (P-BO Padding Mangle, Copower Technology Co., Ltd.) at a pressure of 1 bar. The specimens were then coated with the flame-retardant (FR) solution at a concentration of 100 g/L for 30 s and padded again under the same pressure of 1 bar. After the application of the antistatic agent and flame retardant, the treated nonwoven samples were dried and cured using a stenter machine lab, MingScape International Co., Ltd.) at 120 °C for 20 min. After the finishing process, the aging step was performed at 70 °C for 168 h to simulate long-term thermal exposure and to assess possible volatilization, migration, or degradation of flame-retardant components. The flame-retardant performances of samples before and after aging were evaluated by the UL 94 method.

### 2.6 Characterizations of Treated and Functionalized PF

The diameters of PF samples were observed via an optical microscope (OM, Nikon, Eclipse LV150N). Tenacity and tensile strain profiles of the fibers were measured by the universal testing machine (Instron 5943) according to ASTM D2256 standard (50 N load cell with gauge length and grip separation rates at 250 mm, 300 mm/min, and 50 repeats, respectively). One-way ANOVA followed by Tukey’s HSD post hoc test was used to determine significant differences among the samples for both tenacity and strain. Samples assigned the same letter were considered not significantly different (*p* < 0.05). The surface characteristics of all treated PF were observed by a scanning electron microscope (SEM, Hitachi, SU 8030). Functional groups of PF were analyzed by Fourier transform infrared spectroscopy (Thermo IR-Nicolet iS50). All spectral acquisitions were taken with 64 scans and wavenumbers ranging from 4000 to 400 cm^–1^. The flame-retardant performances of treated PF were investigated via the vertical thin material (VTM) testing (the test sample dimension was 200 × 50 mm for thin and flexible materials) according to the UL 94 standard. The flame-retardant performances of PF nonwoven were investigated via the vertical burning testing (the test sample dimension was 125 × 13 × 13 mm for a self-supporting sample) according to the UL 94 standard. Thermal analysis was performed by thermogravimetric analysis (TGA) (Mettler-Toledo, Model TGA/DSC 3+) under a nitrogen atmosphere (flowing rate at 100 mL/min) with a heating rate of 10 °C/min in the range of 30–800 °C. The sound absorption performance was evaluated according to ASTM E1050–95 standard test method for impedance and absorption of acoustic materials. Washing fastness was assessed according to ISO 105-C10 standards. The surface characteristics of the washing sample were observed by an OM (Nikon, LV100ND LED). The cyclic compression behavior of the nonwoven samples was measured by the mechanical analyzer (Texture Analyzer, Shimadzu EZ-Test, EZ-LX). The test was performed for five compression cycles, which was the maximum allowed by the capacity of the mechanical testing machine, at a constant strain of 50%, with five replicates for each sample. Each cycle has two stages: loading from a small preforce to the strain amplitude and unloading to the preforce. Loading and unloading were performed with a constant crosshead speed of 5 mm/min. Contact angle measurements were performed using an optical contact (LMS Instruments, OCA 15EC). Contact angle measurements were performed three times for each solution on different locations of each surface. Liquids were evaluated in random sequence to avoid the introduction of sampling artifacts.

### 2.7 Sound Absorption Evaluation of PF

The sound absorption coefficient (α) of the samples (staple and nonwoven) was determined using an impedance tube setup following ASTM E1050–95. For the staple fiber samples, thicknesses of 10, 30, and 50 mm were obtained by packing and compressing the fibers under consistent conditions (Supporting Information, Figure S1a–c). For the nonwoven samples, thickness was controlled by stacking layers, where a single layer had a thickness of approximately 10 mm. Accordingly, samples with thicknesses of 30 mm and 50 mm were prepared by stacking 3 layers and 5 layers, respectively (Supporting Information, Figure S1d–f). The impedance tube method enables precise measurement of the absorption coefficient. Alternatively, the NRC offers a simplified, single-value representation of a material’s sound absorption capability. A higher NRC value corresponds to greater sound absorption efficiency of the material. The NRC is calculated using the following formula

$$NRC=\frac{\alpha_{250}+\alpha_{500}+\alpha_{1000}+\alpha_{2000}}{4}$$

where NRC is derived from the average absorption coefficients measured at specific frequencies: 250, 500, 1000, and 2000 Hz.

The sound absorption average (SAA) of a material corresponds to the arithmetic average of its 1/3 octave sound absorption coefficient values in diffuse field, α_*i*_, for the 12 1/3 octave frequency band ranging from 200 to 2500 Hz included: The SAA is calculated using the following formula

$$SAA=\frac{1}{12}\sum_{i=200}^{2500}\alpha_{i}$$

where SAA is derived from the average absorption coefficients measured at specific frequencies: 200, 250, 315, 400, 500, 630, 800, 1000, 1250, 1600, 2000, and 2500 Hz.

## 3 Results and Discussion

### 3.1 Physical and Chemical Characterizations of PF

Physical appearances of PF before and after treatment with enzyme (PF_E) and enzyme-chemical (PF_E_C) are presented in Figure 1. Overall, the physical extracted PF before treatment (Figure 1a) had rougher surface, darker shade, and less uniform fibers than PF_E (Figure 1d) and PF_E_C (Figure 1g). In comparison between treated PF samples, the PF_E_C showed smoother surface and uniform fiber than PF_E. Surface morphology of the PF was further characterized by SEM. Under SEM, the PF revealed large fiber diameter, rough, and irregular surface from appearance of hemicellulose, and other wax contents (Figure 1b).^38,39^ After enzyme treatment, the fibers appeared cleaner than the PF, indicating the removal of some surface impurities such as hemicellulose and lignin (Figure 1e). On the other hand, the PF_E_C showed the smoothest surface and the smallest fiber diameter among three PF samples. This implies that the addition of chemical treatment step further removes amorphous regions and causes fibrillation.^38^ The fiber diameter analysis was evaluated via SEM images followed by quantitative analysis. The result showed that the fiber diameter of PF, PF_E, and PF_E_C was primarily at 83, 65, and 55 μm, respectively (Figure 1c,f,i).

**Figure 1:**
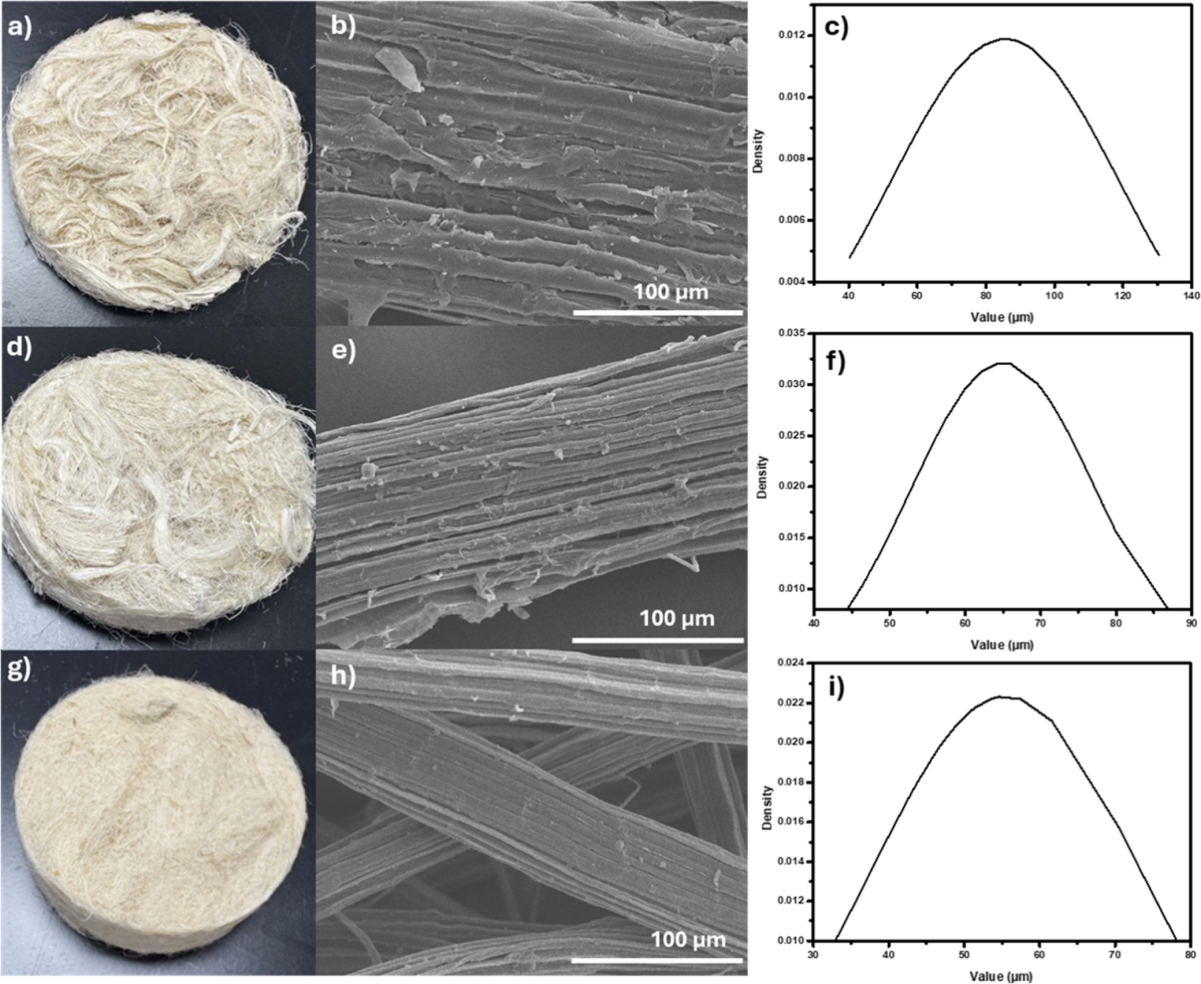
Physical characterizations of PF (a–c) before and after treatment with (d–f) enzyme (PF_E) and (g–i) enzyme-chemical (PF_E_C).

The mechanical properties of PF, PF_E, and PF_E_C were evaluated via a tensile testing machine according to ASTM D2256 standard (Figure 2). The PF exhibited the tenacity and tensile strain at 18.39 cN/tex and 2.78%, respectively. This indicates that the tensile profile of PF from Smooth Cayenne varieties showed approximately among PF from other varieties, such as *Ananas comosus* ^40^ and Sarawakv.^41^ After treatment of the PF with xylanase enzyme (PF_E), the fiber strength reduced to 14.22 cN/tex, while the tensile strain dropped to around 1.39%. This result showed that hemicellulose and lignin were partially removed from the PF. On the other hand, using the combination of xylanase and NaOH for PF treatment (PF_E_C) significantly lowered the tenacity at 8.60 cN/tex and tensile strain at 0.66% of the treated PF. Even though fiber treatment with enzyme-chemical shows the smoothest fiber surface, it greatly impacts the fiber strength, which is one of the important parameters in nonwoven and yarn processing. Fiber strength appears to be closely related to the relative amounts of lignin and hemicellulose present in the fibers. In particular, treatments involving enzyme–chemical methods or cellulase led to a noticeable reduction in the hemicellulose content.^37^ One-way ANOVA followed by Tukey’s HSD post hoc test showed significant differences among the samples in both tenacity and strain (*p* < 0.05). For tenacity, PF and PF_E were statistically similar and assigned the same group, whereas PF_E_C was significantly different. For strain, PF was significantly different, while PF_E and PF_E_C showed no significant difference and were grouped. Therefore, PF_E was selected for the subsequent finishing process with flame-retardant materials as it provided a more suitable balance between surface modification and mechanical integrity.

**Figure 2:**
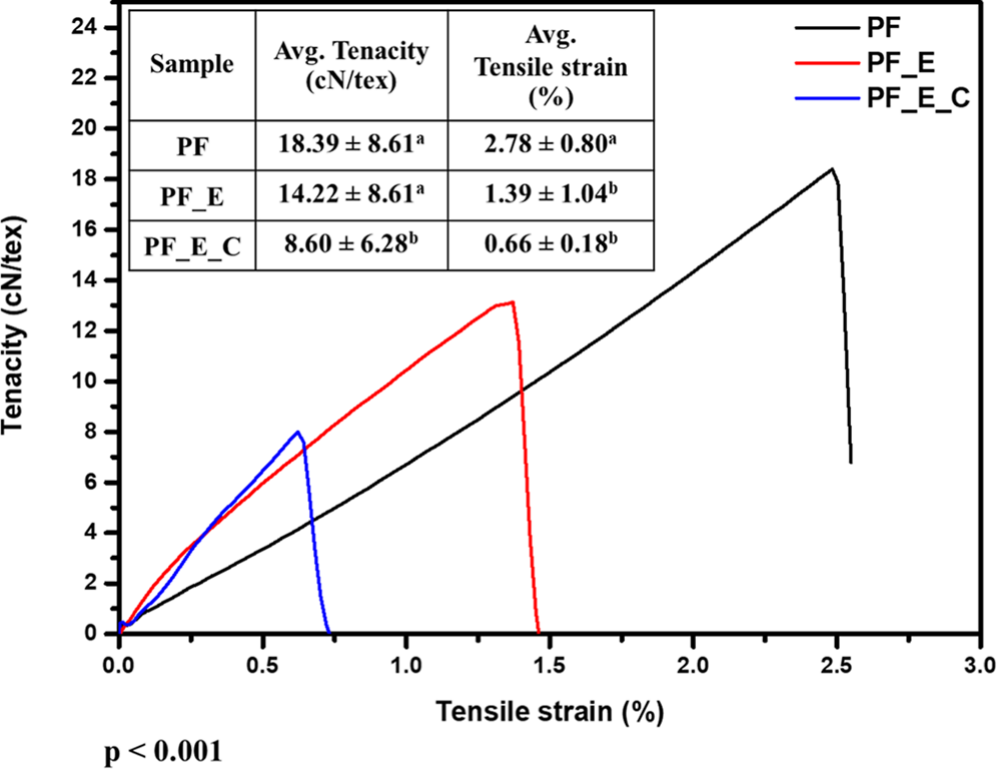
Tensile profiles of PF, PF_E, and PF_E_C.

Two finishing methods with FR and antistatic oil (oil) were finished on PF_E fibers (Supporting Information, Figure S2). In the first procedure, an oil solution at 100 g/L was applied on the PF_E before finishing with an FR solution at 100 g/L, 200 g/L, and 300 g/L concentrations (Figure 3a–f). From images, three finished fiber samples showed similar appearance and colors (Figure 3a,c,e). Under SEM, the finished fiber sample with FR solution at 100 g/L (PF_E_Oil_FR100) showed distributed FR particles on fiber surfaces (Figures 3b and Supporting Information, S3). Finishing the PF_E with FR solution at 200 g/L presented slightly agglomerated FR particles on the fiber surface (Figures 3d and Supporting Information, S4). Increasing the FR solution to 300 g/L during the finishing process resulted in highly agglomerated FR particles on the fiber surface (Figure 3f and Supporting Information, S5). Among the three samples, PF_E_Oil_FR100 exhibited the most favorable surface characteristics with uniform and finely distributed flame-retardant particles. In contrast, PF_E_Oil_FR200 and PF_Oil_FR300 showed flame-retardant clusters on the fiber surface, suggesting that excessive chemical loading leads to nonuniform deposition and potential deterioration of fiber integrity.

**Figure 3:**
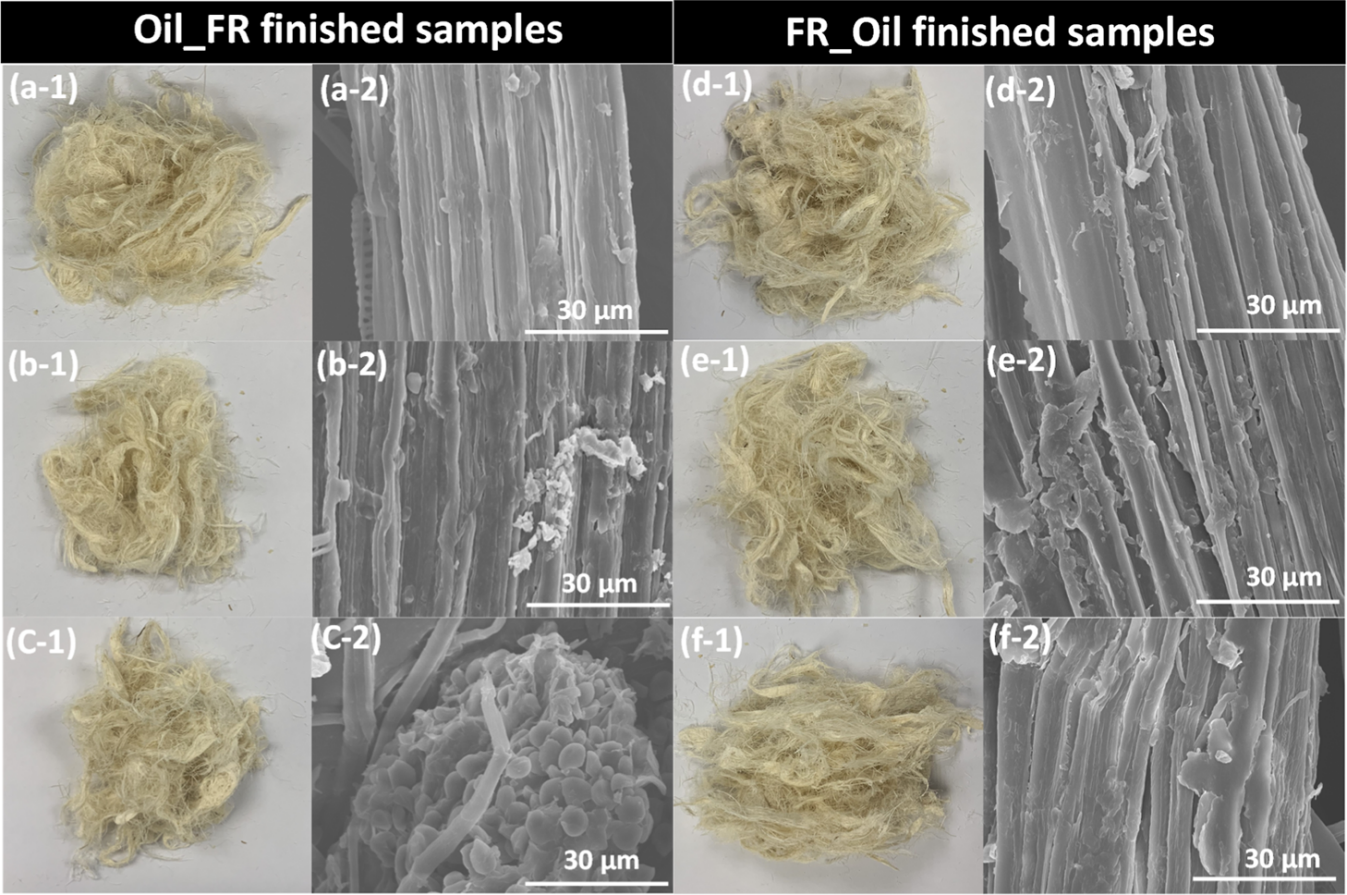
Digital photo and selected SEM images of Oil_FR finished samples of (a-1,a-2) PF_E_Oil_FR100, (b-1,b-2) PF_E_Oil_FR200, (c-1,c-2) PF_E_Oil_FR300, and FR_Oil finished samples of (d-1,d-2) PF_E_FR100_Oil, (e-1,e-2) PF_E_FR200_Oil, and (f-1,f-2) PF_E_FR300_Oil.

In the second route, the PF_E was initially coated with flame-retardant solution at 100 g/L, 200 g/L, and 300 g/L concentrations before applying antistatic oil at 100 g/L concentration. Physical appearances of all fiber samples after finishing showed similar colors (Figure 3g,i,k). Surface morphology of the fibers presented rougher and more irregular surfaces when applying higher concentrations of FR solutions (Figure 3h,j,l). However, fewer FR clusters were observed on the fiber surfaces (FR prior to oil) in comparison to the first finishing procedure (oil prior to FR) (Supporting Information, Figures S6–S8). It could be assumed that applying antistatic oil prior to the flame retardant modifies the fiber surface energy, thereby enhancing the distribution and fixation of FRs.^42^ The significant increase in surface energy after oil treatment from 23 to 78 mN/m (Supporting Information Table S1) indicates improved wettability and surface interaction, which facilitates more uniform distribution and stronger retention of the flame-retardant coating.^43,44^

Investigation of chemical bonding alterations in PF before and after finishing was observed by Fourier-transform infrared spectroscopy (FT-IR) (Figure 4). The PF exhibited characteristic peaks of lignocellulosic fibers (cellulose, hemicellulose, and lignin) such as O–H stretching (∼3300 cm^–1^), C–H stretching (∼2900 cm^–1^), and C–O–C/C–OH stretching (1000–1100 cm^–1^) (Figure 4a).^45^ After treating the PF with enzyme (PF_E), the carbonyl peak intensity at ∼1735 cm^–1^ was significantly reduced, which indicated the partial removal of hemicellulose and lignin contents (Figure 4b).^45^ The treatment of PF with combined enzyme-chemical (PF_E_C) further diminished the carbonyl peak, reflecting increased fiber purity (Figure 4c).^38^

**Figure 4:**
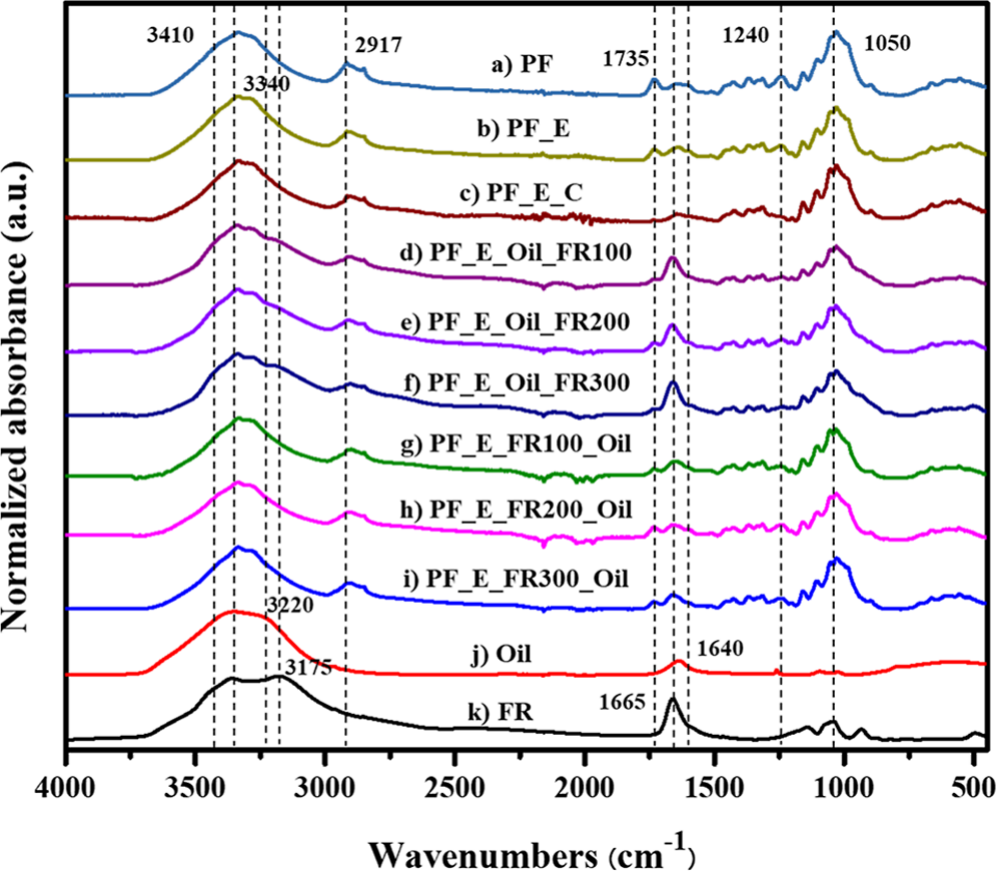
FT-IR spectra of control samples of (a) PF, (b) PF_E, and (c) PF_E_C, Oil_FR finished samples of (d) PF_E_Oil_FR100, (e) PF_E_Oil_FR200, (f) PF_E_Oil_FR300, and FR_Oil finished samples of (g) PF_E_FR100_Oil, (h) PF_E_FR200_Oil, and (i) PF_E_FR300_Oil.

Flame-retardant finished fiber samples (PF_E_Oil_FR100, PF_E_Oil_FR200, and PF_E_Oil_FR300) showed new peaks between 1050 and 1240 cm^–1^, corresponding to P–O–C and P=O functional groups (Figures 4d–f and Supporting Information, Figure S9).^46^ These peaks intensified with higher flame-retardant concentrations. On the other hand, when applying flame retardant before coating with oil, these P–O–C and P=O signals were slightly weaker (Figure 4g–i). This suggested that oil may hinder phosphorus bonding.^47^ Additionally, broader O–H peaks were observed due to increased hydrogen bonding or moisture retention from the hydrophilic oil layer.

The flame-retardant rating of the control and treated PF samples was investigated by VTM testing according to the UL 94 standard (Figure 5). During the testing, the combustion of PF and PF_E was burned completely, leaving only a tiny amount of ash with total after-flame time exceeding 250 s (Figures 5a and Supporting Information S10). This indicated that PF and PF_E showed no flame-retardant performance (no rating). After burning the finished PF with oil before the flame-retardant material, the PF_E_Oil_FR100, PF_E_Oil_FR200, and PF_E_Oil_FR300 showed localized charring and self-extinguishing behavior with minimal after-flame and afterglow times. The total after-flame times of PF_E_Oil_FR100, PF_E_Oil_FR200, and PF_E_Oil_FR300 were 3.98, 5.49, and 5.80 s, respectively (Table 1). In addition, charring remained consistent despite the increased flame-retardant loading, suggesting that the flame-retardant content did not affect flammability. Therefore, all of the samples demonstrated significant flame resistance and achieved the UL 94 standard at V-0 rating.^48^ In contrast, PF_E samples that were treated with flame retardant prior oil to the material (PF_E_FR100_Oil, PF_E_FR200_Oil, and PF_E_FR300_Oil) did not pass any UL 94 rating (Figure 5e–g). The total after-flame time of PF_E_FR100_Oil, PF_E_FR200_Oil, and PF_E_FR300_Oil exceeded 250 s with the after-flame of all the samples extending to 125 mm. It can be hypothesized that applying antistatic oil after FR materials suppresses performance of FR, which results in significant combustion and structural deterioration. Among all finished PF samples, PF_E_Oil_FR100 achieved the optimal balance in functional performance, morphological stability of additives, and cost effectiveness.

**Figure 5:**
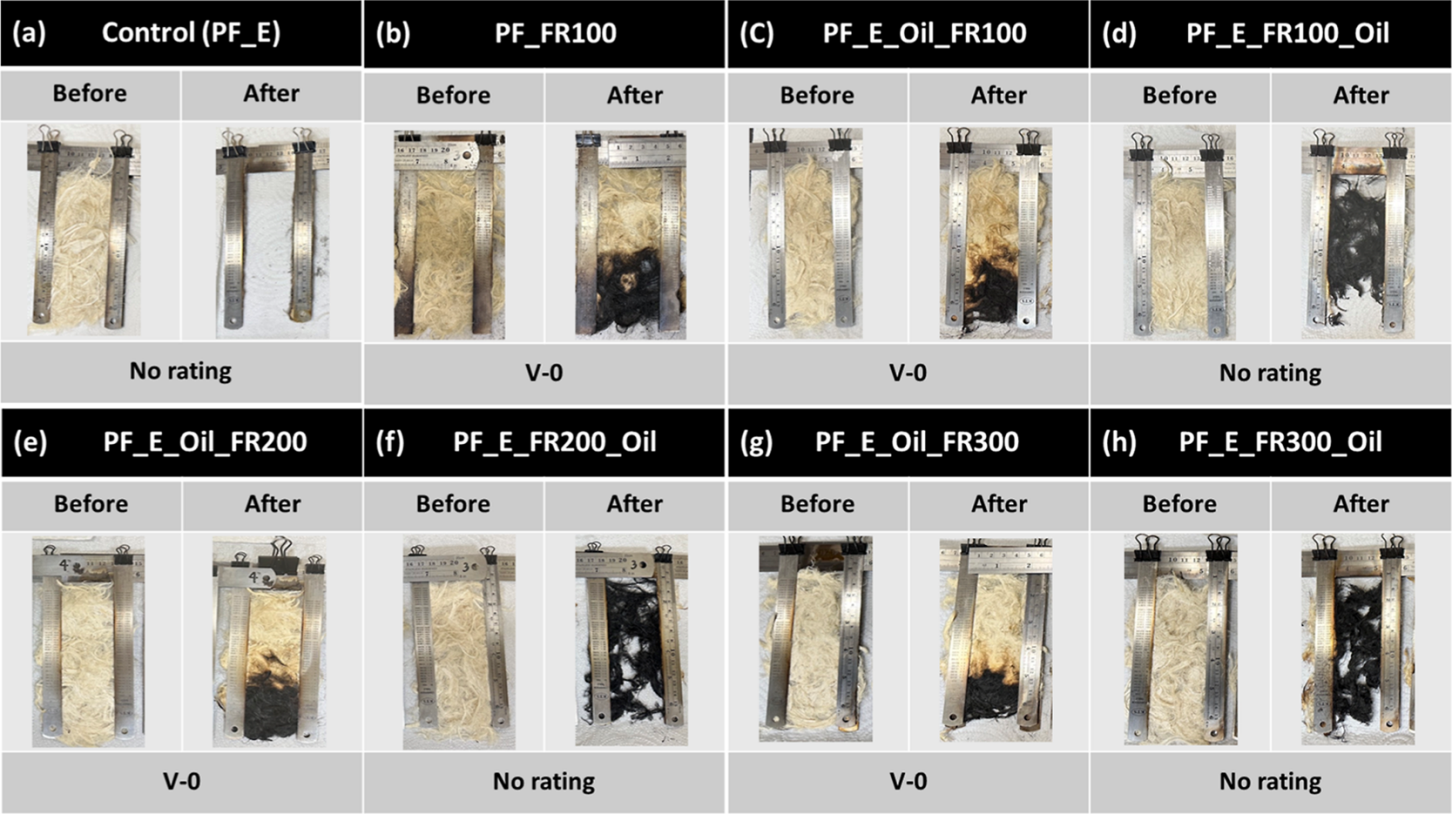
Flame-retardant properties of control samples of (a) PF_E, (b) PF_E_FR100, Oil_FR finished samples of (c) PF_E_Oil_FR100, (d) PF_E_Oil_FR200, (e) PF_E_Oil_FR300, FR_Oil finished samples of (f) PF_E_FR100_Oil, (g) PF_E_FR200_Oil, and (h) PF_E_FR300_Oil (0.5 bar).

**Table 1:** Flame-Retardant Properties of the Other PF and Commercial Samples (VTM)

sample	T1 (5 repeats) (s)	T2 (5 repeats) (s)	time total (T1 + T2) (s)	avg. burning length (%)
PF	>50	>50	>600	100
PF_E	>50	>50	>600	100
PF_E_Oil_FR100	1.94	2.04	3.98	40.56 ± 2.23
PF_E_Oil_FR200	2.11	3.38	5.49	46.01 ± 9.05
PF_E_ Oil_FR300	2.52	3.28	5.80	40.40 ± 6.13
PF_E_FR100 _Oil	>50	>50	>600	100
PF_E_ FR200 _Oil	>30	>30	>250	79.55 ± 26.43
PF_E_ FR300 _Oil	>30	>30	>250	100
PF_FR100	3.97	4.53	8.50	47.11 ± 6.46
PF_Oil_FR100	1.52	1.87	3.39	45.10 ± 5.16
PF_E_FR100	3.29	4.29	7.58	50.94 ± 28.44
80PF_E_20LM_Oil_FR100	6.18	4.35	10.53	35.22 ± 6.18

In addition to flame-retardant testing of enzyme-treated PF samples, the other PF samples and commercial material containing PF with low-melt PET for nonwoven production were also evaluated (Table 1 and Supporting Information S10). The burned lengths of PF_FR100 and PF_E_FR100 showed localized charring and self-extinguishing behavior and achieved the UL 94 standard at V-0 rating (Table 1). It could be concluded that addition of oil in the finishing process did not affect the flame-retardant properties of the PF samples. The commercial nonwoven treated with oil and FR (80PF_E_20LM_Oil_FR100) exhibited the total after-flame time and percentage burning length of 10.53 s and 35.22%, respectively. Therefore, the finishing conditions with PF_E could be applied with the commercial 80PF_E_20LM nonwoven materials.

The flame-retardant performance of PF_E_Oil_FR100 strongly depended on the padding pressure, as shown by the variation in wet pickup and the UL-94 results (Table 2). At lower padding pressures (0.5–2.5 bar), the samples exhibited relatively high wet pick-up values, which enabled sufficient uptake of the flame-retardant formulation and resulted in successful UL-94 performance both before and after aging. In contrast, increasing the padding pressure to 3.5 and 4.5 bar substantially reduced wet pickup, indicating excessive removal of the finishing solution from the fibrous structure. This reduction in retained flame-retardant content led to inadequate flame inhibition and char formation, causing the samples to fail the UL-94 test regardless of the aging treatment.

**Table 2:** Flame-Retardant Results after Aging of PF_E_Oil_FR100 at Pressures of 0.5, 1.5, 2.5, 3.5, and 4.5 bar

		UL 94 result (PF_E_Oil_FR100)	
pressure (bar)	avg. wet pick-up (%)	before aging	after aging
0.5	107.46 ± 5.59	pass (V-0)	pass (V-0)
1.5	83.36 ± 2.73	pass (V-0)	pass (V-0)
2.5	72.02 ± 1.58	pass (V-0)	pass (V-0)
3.5	63.92 ± 3.94	false (V-1)	false (no rating)
4.5	57.46 ± 6.51	false (V-1)	false (no rating)

The further experimental variations of chemical pick-up percentage of PF_E_Oil_FR100 after aging at 70 °C for 168 h were performed to optimize the finishing conditions. To optimize the percentage of wet pick-up, the compression pressure of the padding process was systematically varied (Table 2 and Figure 6). The results indicated that the total after-flame times for PF_E_FR100_Oil after padding with a pressure of 0.5, 1.5, and 2.5 bar were 14.01, 39.05, and 41.60 s, respectively. These samples exhibited localized charring and self-extinguishing behavior, with minimal after-flame and afterglow durations. Consequently, all samples demonstrated substantial flame resistance and met the UL 94 V-0 classification. However, finishing process with a pressure of 3.5 and 4.5 bar resulted in the total after-flame time that exceeded 250 s, and the flame propagation extended up to 125 mm (no rating). It could be hypothesized that inconsistent charring was observed when the flame-retardant content was reduced (3.5 and 4.5 bar), suggesting that the quantity of flame-retardant material significantly influences flammability.

**Figure 6:**
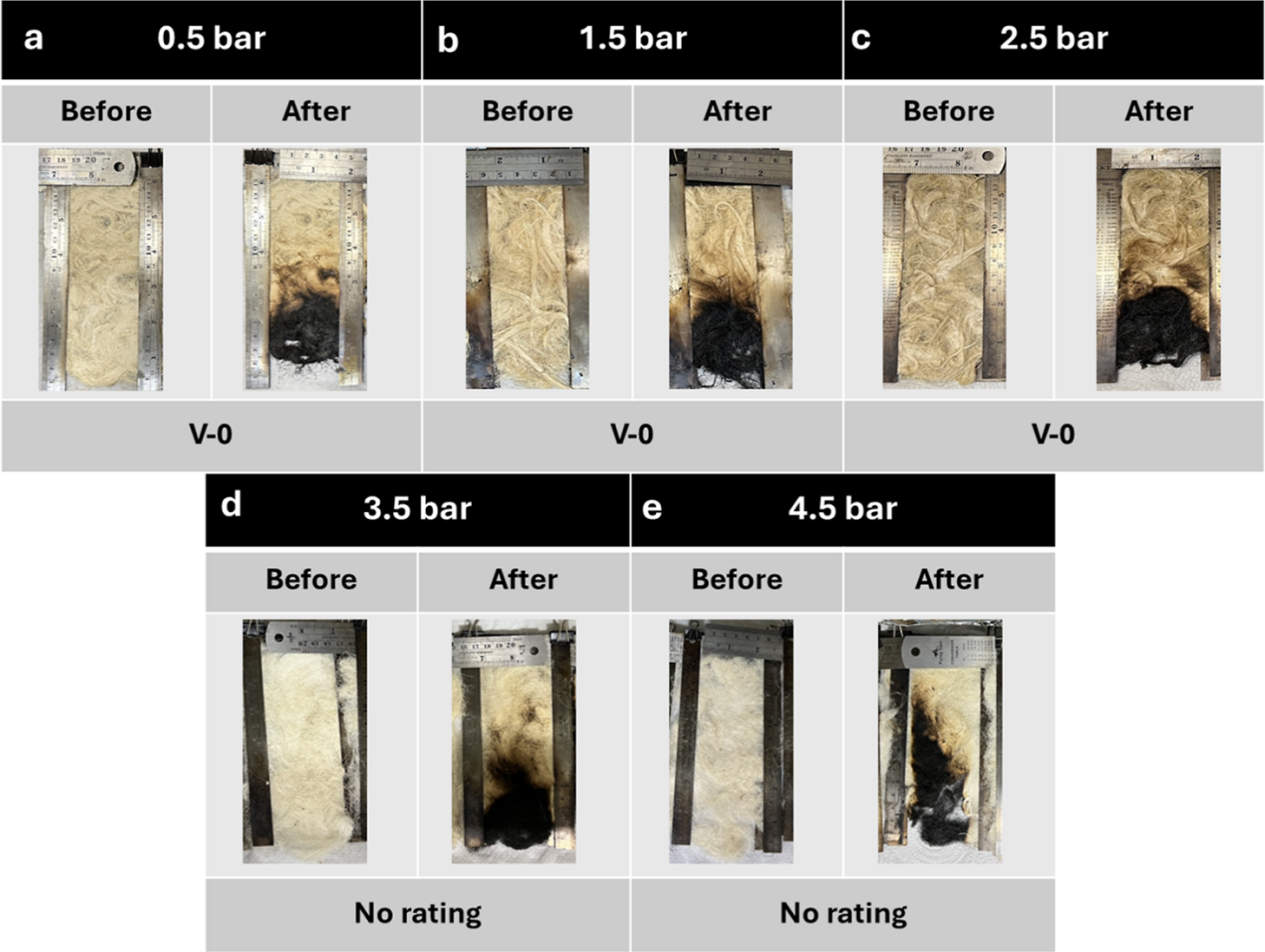
Flame-retardant properties after 2nd ignition and aging of PF_E_Oil_FR100 at pressure of (a) 0.5, (b) 1.5, (c) 2.5, (d) 3.5, and (e) 4.5 bar.

The flame-retardant performance of the 80PF_E_20LM nonwoven was assessed before and after aging, as presented in Table 3 and Figure 7. The untreated samples (80PF_E_20LM_NW and 80PF_E_20LM_NW_A) showed a very poor fire resistance. In both cases, the total after-flame time exceeded 500 s (Table 3), and during the second ignition test, the samples burned completely and were reduced to full char (Figure 7a,b). As a result, untreated materials satisfied the UL-94 rating requirements, demonstrating that the 80PF_E_20LM nonwoven alone does not provide sufficient flame protection for pineapple fiber nonwoven materials. In contrast to the treated samples, adding the optimized FR formulation (Oil_FR100) greatly improved the flame-retardant properties of the nonwoven material. The nonaged sample (80PF_E_20LM_oil_FR100_NW) exhibited the total after-flame time and percentage burning length of 15.80 s and 37.24%, respectively (Table 3). The 80PF_E_20LM_oil_FR100_NW showed self-extinguishing after ignition and only minimal char formation, achieving a UL-94 V-0 rating. This response is consistent with the action of phosphorus-based flame retardants, which promotes dehydration and helps stabilize char within lignocellulosic fibers. Even after accelerated aging, the treated sample (80PF_E_20LM_oil_FR100_NW_A) continued to perform well, maintaining its structure and preserving the UL-94 V-0 rating. These results highlight the durability of the FR system and suggest that the phosphorus-based chemistry remained strongly bonded to the PF fibers with no significant loss or degradation during the aging process. To further evaluate the stability of the flame-retardant coating, a washing test was additionally performed, followed by OM observation (Supporting Information, Figure S12). The OM images before washing clearly show the distribution of the flame-retardant coating on the fiber surface. After washing, although part of the coating layer was removed, some coating residues remained attached to the fiber surface, suggesting that the FRs were at least partially retained through physical adsorption. These results indicate partial retention and acceptable durability of the flame-retardant treatment after washing.

**Table 3:** Flame-Retardant Properties of the Other PF and Commercial Samples (V0 V1 V2)

sample	T1 (5 repeats) (s)	T2 (5 repeats) (s)	time total (T1 + T2) (s)	avg. burning length (%)
80PF_E_20LM_NW	>500	0	>500	100
80PF_E_20LM_Oil_FR100_NW	0	15.80	15.80	37.24 ± 8.89
80PF_E_20LM_NW_A	>500	0	>500	100
80PF_E_20LM_Oil_FR100_NW_A	2.80	39.50	42.30	43.44 ± 4.74

**Figure 7:**
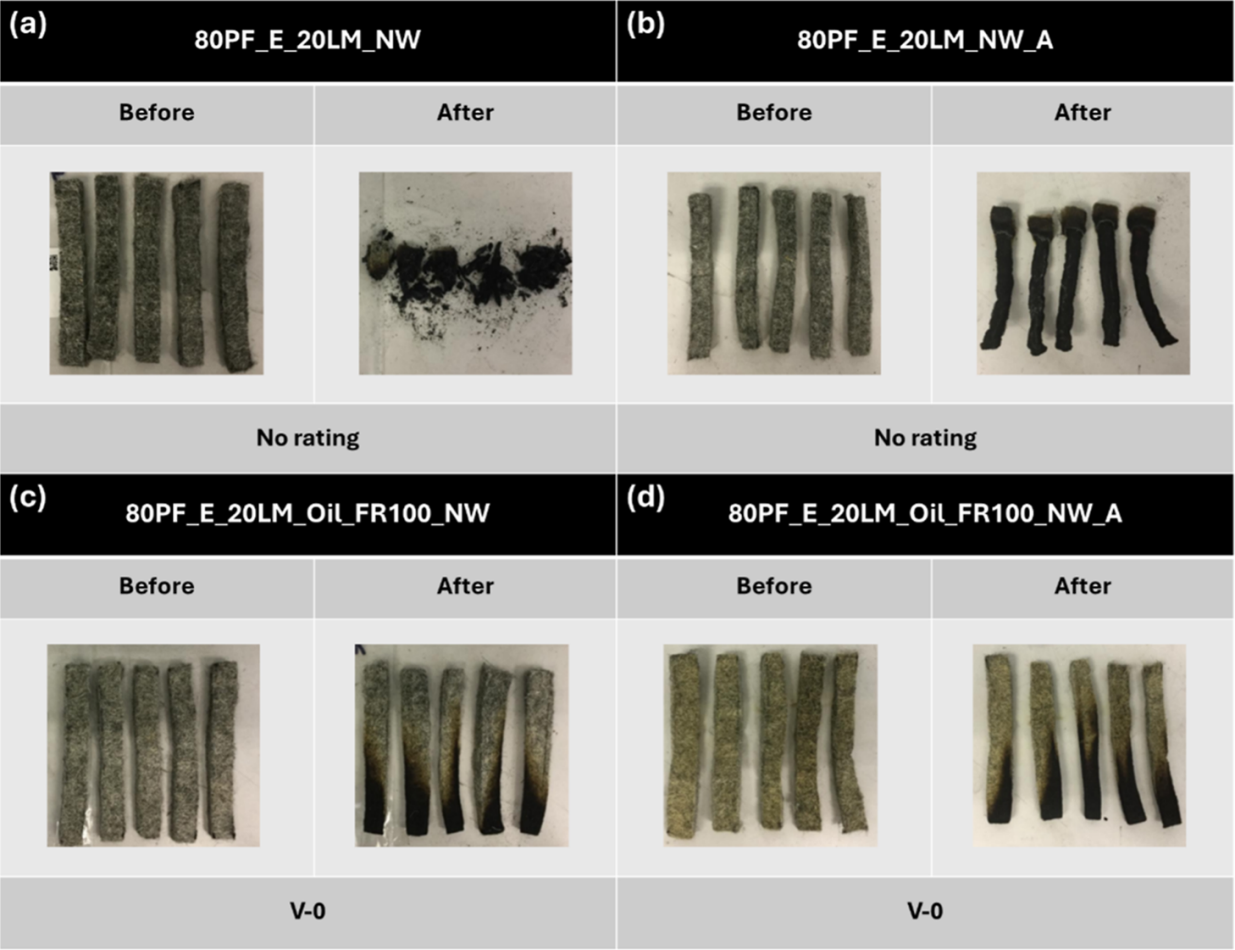
Flame-retardant properties after the second ignition of nonwoven before and after aging (a) 80PF_E_20LM_NW before aging, (b) 80PF_E_20LM_NW after aging, (c) 80PF_E_20LM_Oil_FR100_NW before aging, and (d) 80PF_E_20LM_Oil_FR100_NW after aging.

TGA of PF, PF_E_Oil_FR100, and PF_E_FR100_Oil was performed to investigate the thermal stability and degradation characteristics of the finished PF samples with different finishing sequences (Figures 8 and Supporting Information, S13). The PF exhibited a typical two-step thermal degradation pattern. The major weight losses started at ∼280 °C and continued to ∼400 °C, followed by the formation of a char residue (∼19%) at ∼800 °C. The TGA of PF_E_Oil_FR100 showed increasing in the initial degradation temperature and char residue (∼23%) which indicate enhanced thermal stability. For PF_E_FR100_Oil, the TGA showed an earlier degradation onset around 270 °C and a highly residual weight (∼31%) at 800 °C. The initial weight loss started at ∼280–300 °C attributed to the breakdown of hemicellulose and the initial stages of lignin decomposition.^49^ The lower onset degradation temperature of the finished PF was mostly because phosphorus-based flame retardants degrade into phosphoric acid and polyphosphoric acid at a lower temperature, thus promoting fiber dehydration to produce a stable carbon structure.^48^ The increased char production of PF_E_FR100_Oil was possibly due to incomplete combustion or the formation of a thermally stable oil-derived residue, rather than effective flame retardancy.^50^ The generated char layer may have lacked sufficient integrity and compactness to effectively block heat transfer, oxygen diffusion, and volatile release during combustion, as previously reported for phosphorus-based flame-retardant systems.^51^ Previous studies have reported that flame-retardant performance depends not only on the amount of char residue formed during combustion but also on the compactness and protective ability of the resulting char layer, where a continuous char structure can effectively reduce heat and mass transfer.^52^

**Figure 8:**
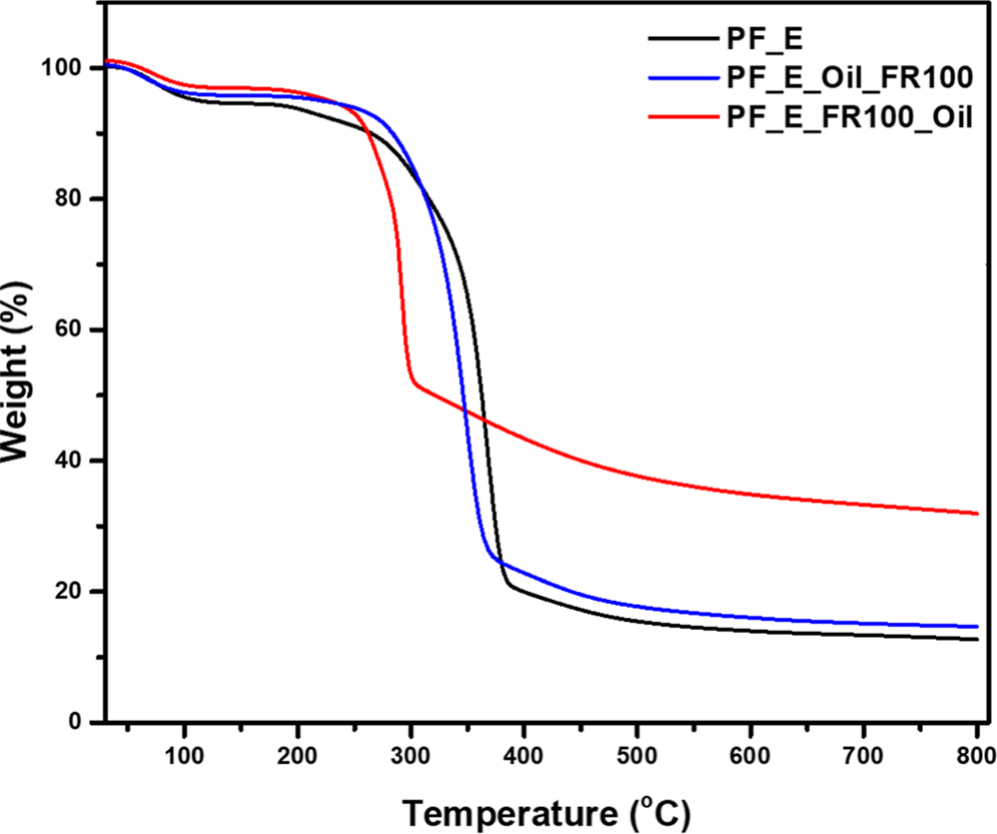
TGA of (a) PF_E, (b) PF_E_Oil_FR100, and (c) PF_E_FR100_Oil.

The acoustic properties of PF_E_Oil_FR100 were evaluated via NRC testing in comparison with PF_E and commercial PF (PF_Com) at sample thickness of 10, 30, and 50 mm (Table 4 and Supporting Information Table S3). At 10 mm thickness, all three samples exhibited identical NRC values at 0.15, indicating limited sound absorption at low thickness. The SAA values of commercial PF showed the highest value at 0.22, while PF_E and PF_E_Oil_FR100 presented the value at 0.15. This can be hypothesized that enzyme treatment and finishing processes on PF reduce porosities and hollow microstructure of the PF.^38^ As the sample thickness increased to 30 mm, NRC of all samples increased to 0.40, while the SAA values of PF_E and PF_E_Oil_FR100 were 0.37 and 0.41, respectively. At the thickest sample size (50 mm), the NRC of PF_Com presented the highest value at 0.65, while PF_E and PF_E_Oil_FR100 samples showed the value at 0.55. On the other hand, the SAA values of all three samples were almost at the identical value between 0.55 and 0.57. In the low-and mid-low-frequency regions, the sound absorption coefficient increased, as influenced by the available fiber surface area and surface morphology. The treated fibers (PF_E) exhibited a relatively smooth surface due to the partial removal of hemicellulose and lignin during the extraction process. In contrast, the finished fibers (PF_E_Oil_FR100) showed a rougher surface after the coating treatment. The increased surface roughness likely promoted greater interaction between the incident sound waves and the fiber surface, leading to the improved sound absorption performance.^34,53^ The overall sound absorption of samples increased because a thicker porous layer provides a longer dissipation path, enhancing viscous and thermal losses within the fibrous network. This could be concluded that PF_E_Oil_FR100 was able to utilize as a natural flame-retarded sound absorption material. A clear improvement in sound absorption was observed when the pineapple fibers were converted into nonwoven materials. At a thickness of 50 mm, both 80PF_E_20LM_NW and 80PF_E_20LM_Oil_FR100_NW showed higher NRC and SAA values than the loose fibers. This trend agrees with previous work on PFs and their assemblies, where controlling fiber packing, density, and structure (such as nonwoven mats or layered fabrics) was shown to enhance the absorption coefficient, especially at mid-high frequencies.^22,54^ In the nonwoven form, the fibers create a more complex pore network and higher airflow resistivity, which increases viscous and thermal losses when sound waves travel through the material. Similar behavior has been reported for pineapple-fiber nonwovens and other needle-punched nonwoven acoustic panels.^55,56^ Importantly, the FR-treated nonwoven (80PF_E_20LM_Oil_FR100_NW) maintained strong acoustic performance, indicating that the flame-retardant finishing did not significantly block the pores or stiffen the structure. This agrees with studies on natural-fiber acoustic panels, where suitable surface or binder systems were found to improve fire performance while preserving the porous morphology needed for sound absorption.^57^ The mechanical behavior of the nonwoven samples (80PF_E_20LM_NW and 80PF_E_20LM_Oil_FR100_NW) remained relatively stable over multiple loading cycles (Supporting Information, Figure S14 and Table S2). The treated nonwoven (80PF_E_20LM_Oil_FR100_NW) exhibited higher maximum stress compared to the untreated sample (80PF_E_20LM_NW), indicating increased stiffness due to the oil and flame-retardant treatment.^58–60^ These results demonstrate that the finished nonwoven materials possess suitable mechanical durability for potential application in textile industrial production. Taken together, these results suggest that the FR-modified pineapple-fiber nonwovens can provide both effective sound absorption and improved fire safety, making them promising for applications such as interior panels, natural flame-retardant sound-absorbing materials, and automotive acoustic components.

**Table 4:** Sound Absorption Test of Commercial PFs (PF_Com), PF_E, and PF_E_Oil_FR100

		NRC			SAA			
		sample thickness (mm)			sample thickness (mm)			
sample	density (g/cm^3^)	10	30	50	10	30	50	Refs
PF_Com	0.089	0.15	0.40	0.65	0.22	0.43	0.57	^37^
PF_E	0.090	0.15	0.40	0.55	0.15	0.37	0.55	^37^
PF_E_Oil_FR100	0.090	0.15	0.40	0.55	0.15	0.41	0.57	this work
80PF_E_20LM_NW	0.157	0.25	0.55	0.60	0.23	0.54	0.62	this work
80PF_E_20LM_Oil_FR100_NW	0.199	0.20	0.60	0.65	0.21	0.57	0.62	this work

## 4 Conclusions

This study investigates the surface modification of PFs and their utilizations as flame-retarded sound absorption materials. The fibers were extracted using two distinct approaches, a sole enzymatic (PF_E) and a combined enzyme–chemical (PF_E_C) treatments, to purify fibers and facilitate efficient separation. The PF treatment with enzyme-chemical showed the smoothest fiber surface, but the treatment processes greatly reduced the fiber strength. The finishing methods with flame-retardant agent (FR) and antistatic oil (oil) were experimented on PF_E fibers. It was observed that finishing the treated fibers with oil prior to FR demonstrated superior flame resistance properties that is able to achieve a V-0 rating in UL 94 standard testing. The optimized finishing process in terms of flame resistance and cost effectiveness was using FR at 100 g/L at a padding pressure of 1.5 bar with %wet pick-up of approximately 83%. It could be concluded that the functional finishing on treated PF did not deteriorate the acoustic performance compared to nontreated one. Moreover, the sound absorption coefficient of FR-treated nonwovens was significantly improved at lower frequencies (<2000 Hz) when increasing the sample thickness. Additionally, the compression resilience properties of the FR-treated PF nonwoven were superior to those of the untreated sample. Therefore, the enzyme-treated PF with flame-retardant finishing (PF_E_Oil_FR100) could be applied in textile industrial production and utilized as environmentally friendly flame-retardant sound-absorbing material.

## Supplementary Material



## Notes

Sample size and impedance tube equipment for sound absorption testing according to ASTM E1050–95; morphological analysis and element mapping of the finishing sample; surface energy of nonwoven; FTIR of finishing agent; flame-retardant properties; DTG of staple fiber; OM images; TGA and DTG of finishing agents; curves of stress–strain at 50% strain amplitude under 5 compression cycles; stress at max and energy absorption; and the sound absorption characteristic of all sample (PDF)
