# Supplementary material for: Flame-Retardant-Functionalized Pineapple Leaf Fibers for Sustainable Acoustic Absorption
Source: ACS Omega. 2026 Jun 24;11(26):39090–101. doi: 10.1021/acsomega.6c02520 (PMC13347324; doi:10.1021/acsomega.6c02520)
Supplement: Supplementary file 1 [file ao6c02520_si_001.pdf]

Supporting Information

Flame Retardant Functionalized Pineapple Leaf Fibers for Sustainable Acoustic Absorption

Sunisa Suwatthi[a], Kritsana Janyajaraskul[a], Jitlada Boonlertsamut[a], Nakarin Subjalearndee[a], Varol Intasanta[b*], Chutima Vanichvattanadecha[a*]

[a] Advanced Composite and Nanotextiles Research Team, National Nanotechnology Center, National Science and Technology Development Agency, Pathum Thani 12120, Thailand

[b]Chemistry Department, Faculty of Science, Chulalongkorn University, Bangkok 10330, Thailand

*Corresponding author: Dr. Chutima Vanichvattanadecha

Tel: +66 2 5646500, Email: Chutima.van@nanotec.or.th

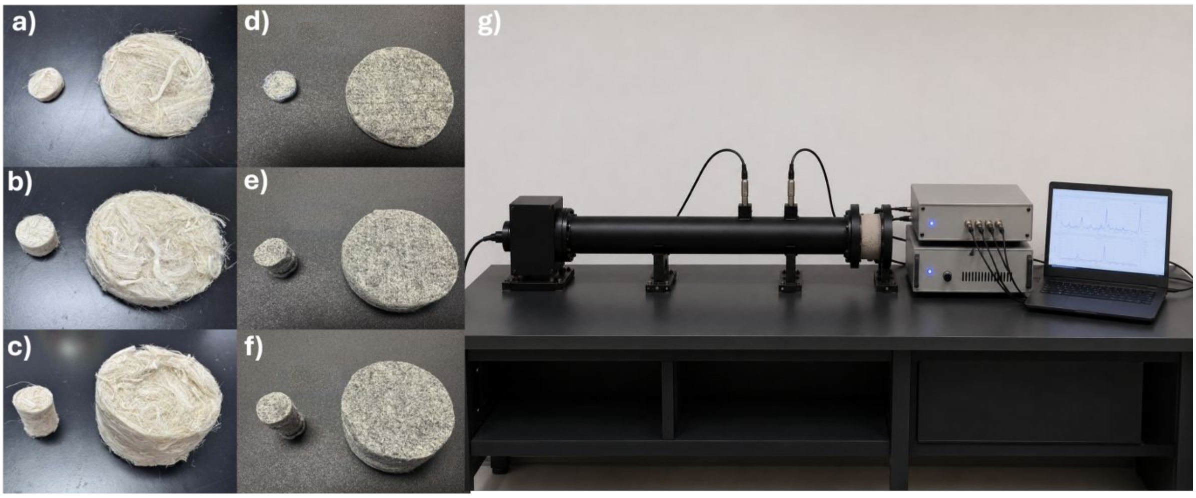

Figure S1. Sample size of a) PF_E staple fiber (1 cm), b) PF staple fiber (3 cm), c) PF staple fiber (5 cm), d) flame-retarded PF_PET non-woven (1 cm), e) flame-retarded PF_PET non-woven (3 cm), f) flame-retarded PF_PET non-woven (5 cm), and g) impedance tube equipment for sound absorption testing according to ASTM E1050-95.

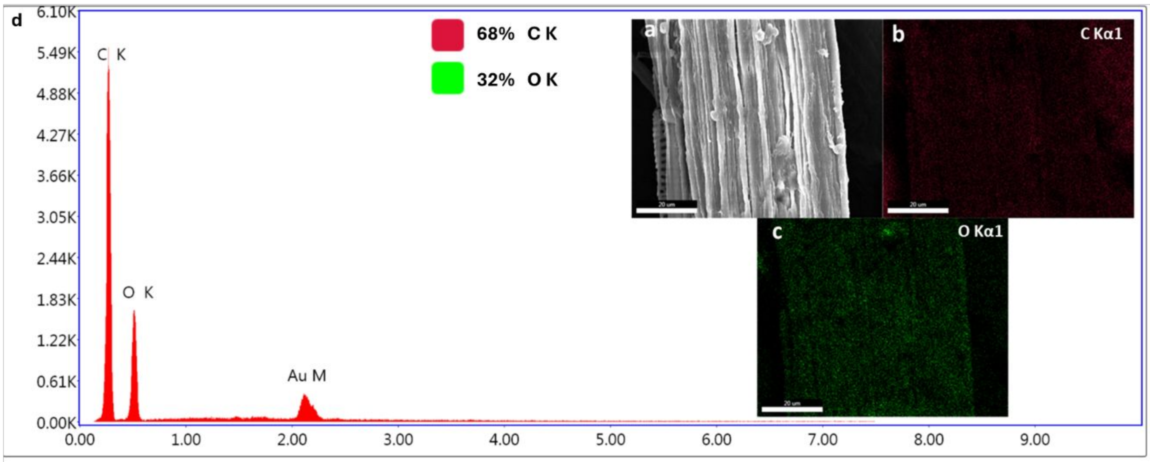

Figure S2. Morphological analysis and element mapping of PF_E a) SEM image, b-c) SEM-EDX element mapping, and d) SEM-EDX spectrum.

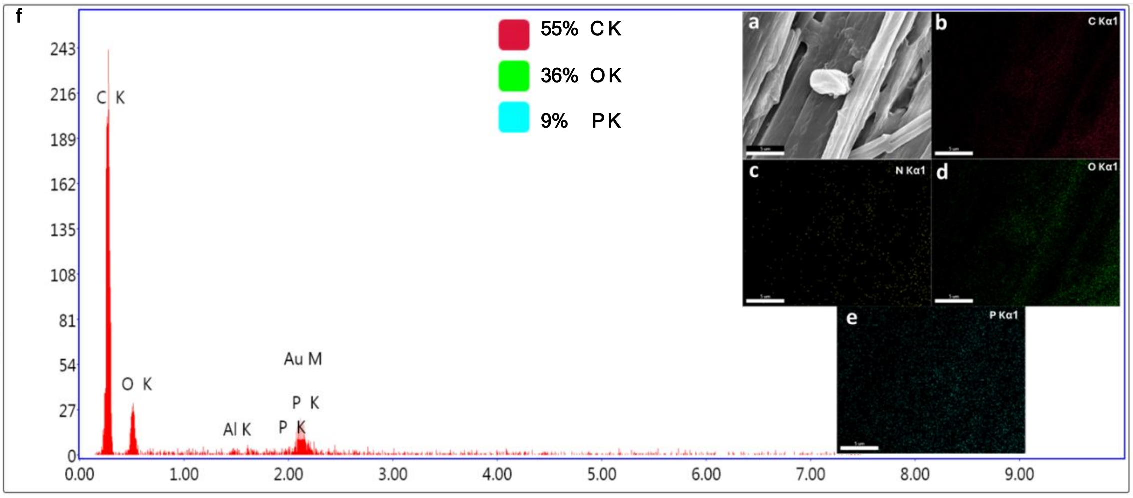

Figure S3. Morphological analysis and element mapping of PF_E_Oil_FR100 a) SEM image, b-e) SEM-EDX element mapping, and f) SEM-EDX spectrum.

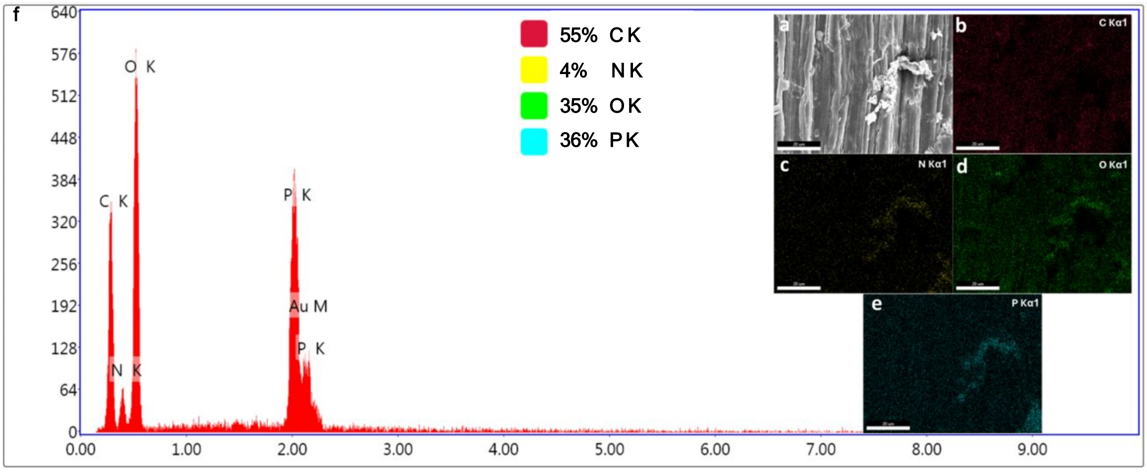

Figure S4. Morphological analysis and element mapping of PF_E_Oil_FR200 a) SEM image, b-e) SEM-EDX element mapping, and f) SEM-EDX spectrum.

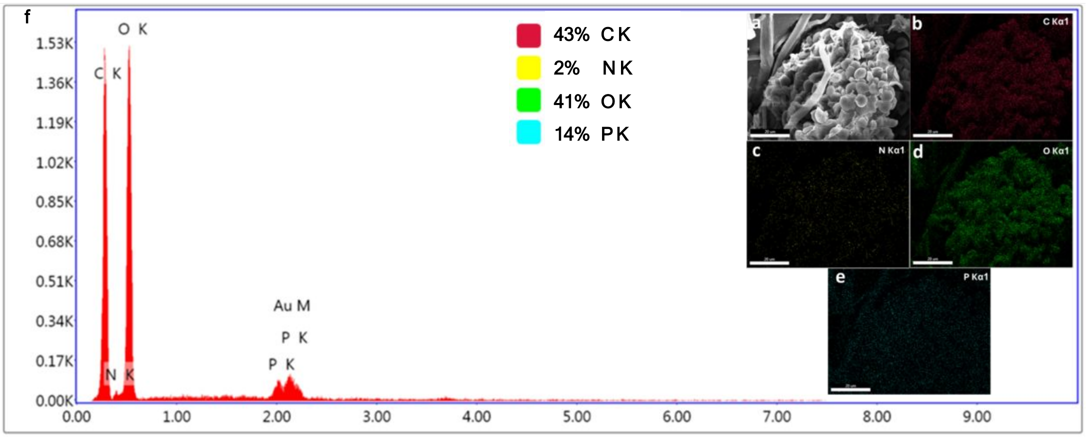

Figure S5. Morphological analysis and element mapping of PF_E_Oil_FR300 a) SEM image, b-e) SEM-EDX element mapping, and f) SEM-EDX spectrum.

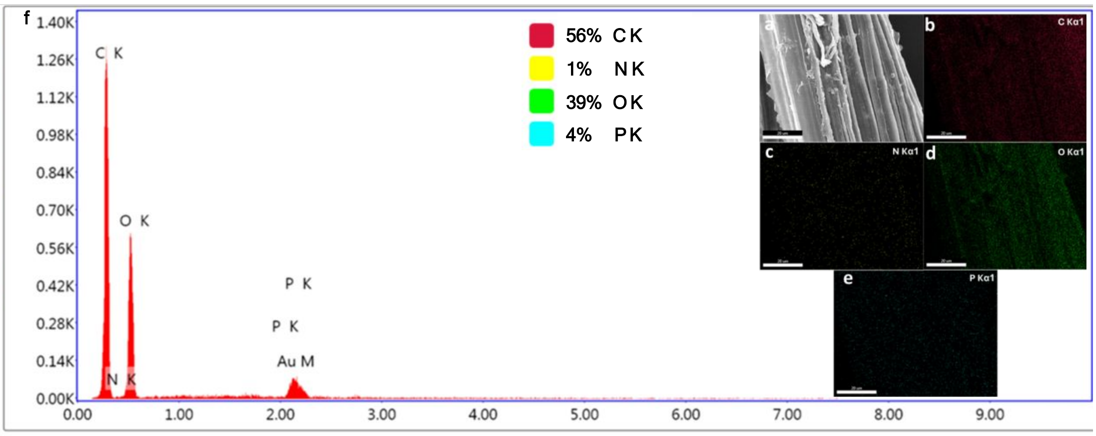

Figure S6. Morphological analysis and element mapping of PF_E_FR100_Oil a) SEM image, b-e) SEM-EDX element mapping and f) SEM-EDX spectrum.

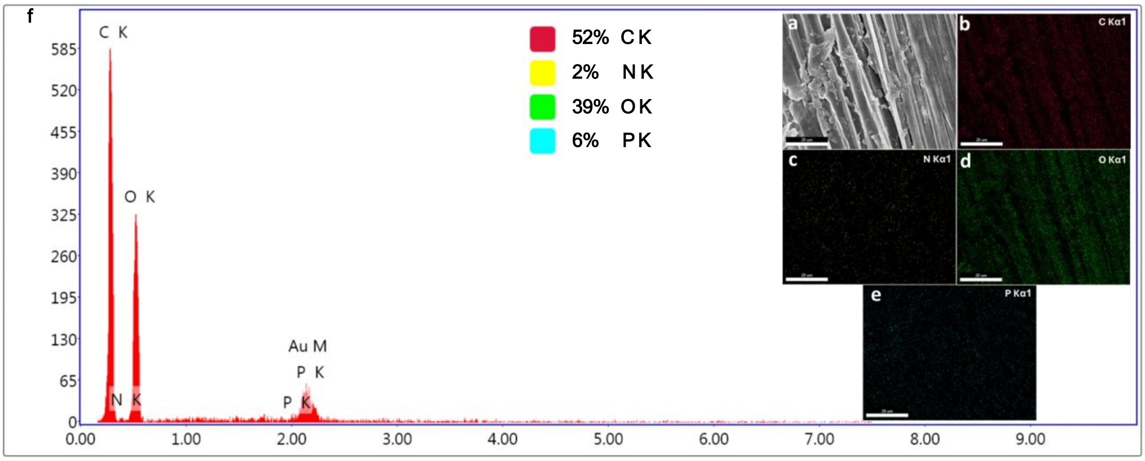

Figure S7. Morphological analysis and element mapping of PF_E_FR200_Oil a) SEM image, b-e) SEM-EDX element mapping and f) SEM-EDX spectrum.

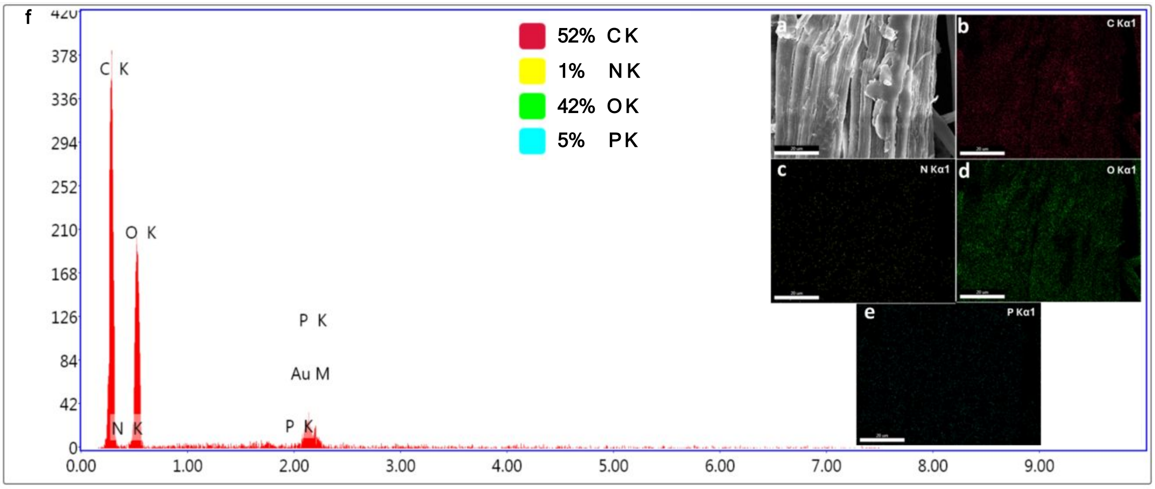

Figure S8. Morphological analysis and element mapping of PF_E_FR300_Oil a) SEM image, b-e) SEM-EDX element mapping and f) SEM-EDX spectrum.

Table S1. Surface energy of 80PF_E_20LM_NW, 80PF_E_20LM_Oil_NW, and 80PF_E_20LM_Oil_FR100_NW

Sample	Surface Energy (mN/m)
80PF_E_20LM_NW	23.33 ± 0.96
80PF_E_20LM_NW_FR	67.42 ± 0.96
80PF_E_20LM_Oil_NW	78.02 ± 0.94
80PF_E_20LM_Oil_FR100_NW	79.07 ± 0.98

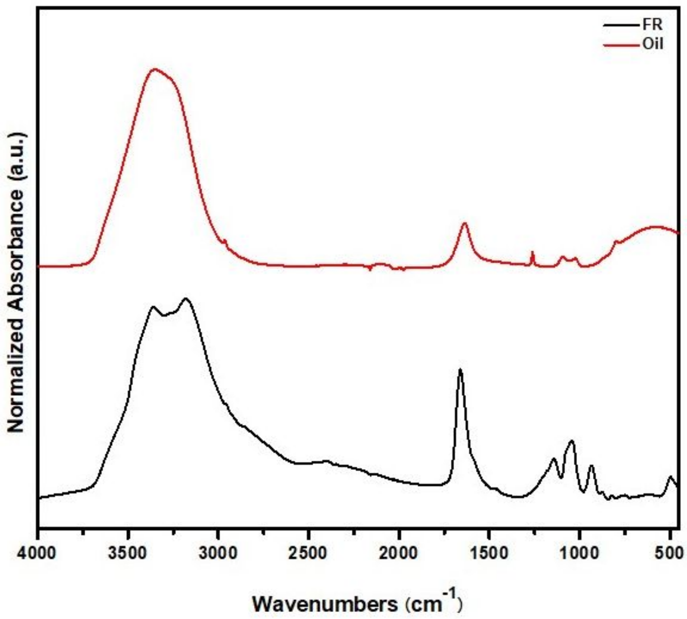

Figure S9. FT-IR of FR and Oil.

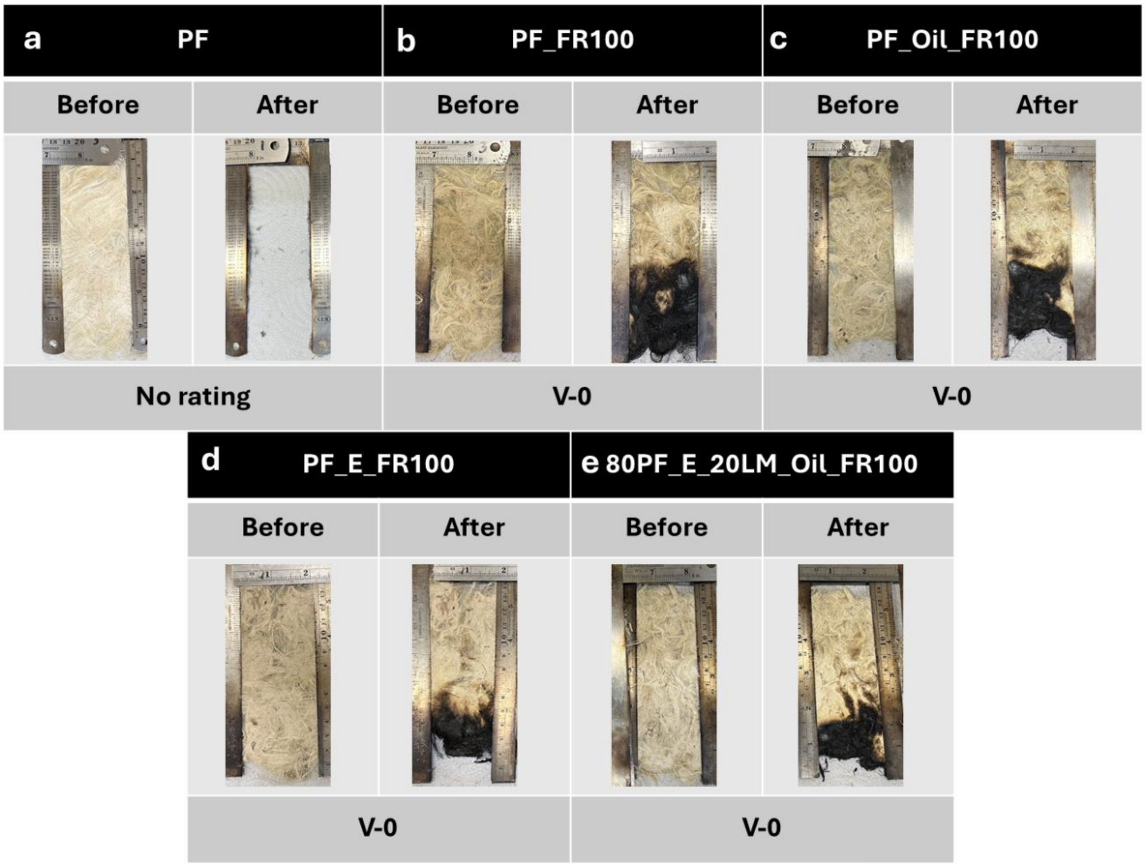

Figure S10. Flame retardant properties of PF, PF_FR100, PF_Oil_FR100, PF_E_FR100, and 80PF_E_20LM_Oil_FR100.

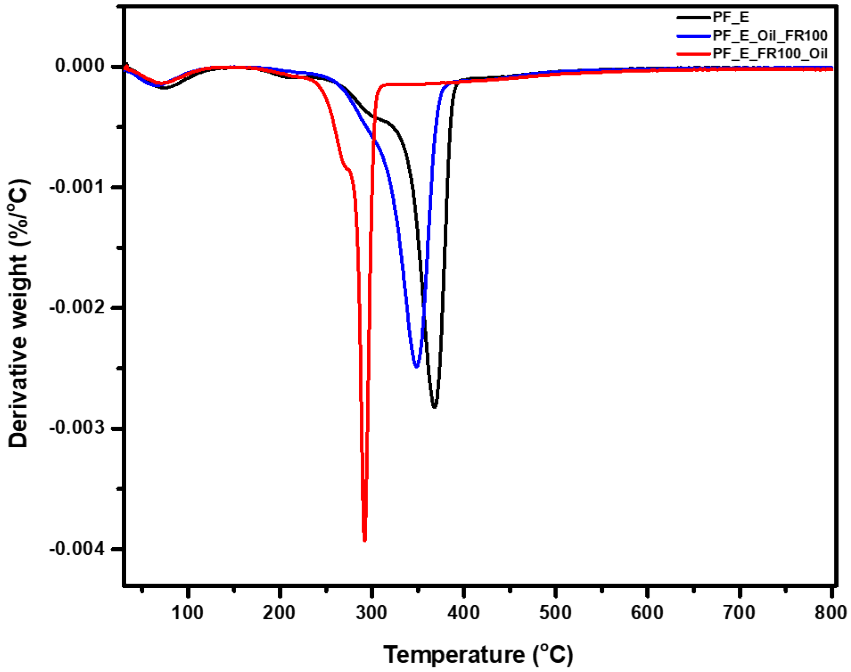

Figure S11. DTG of PF_E, PE_E_Oil_FR100, and PE_E_FR100_Oil.

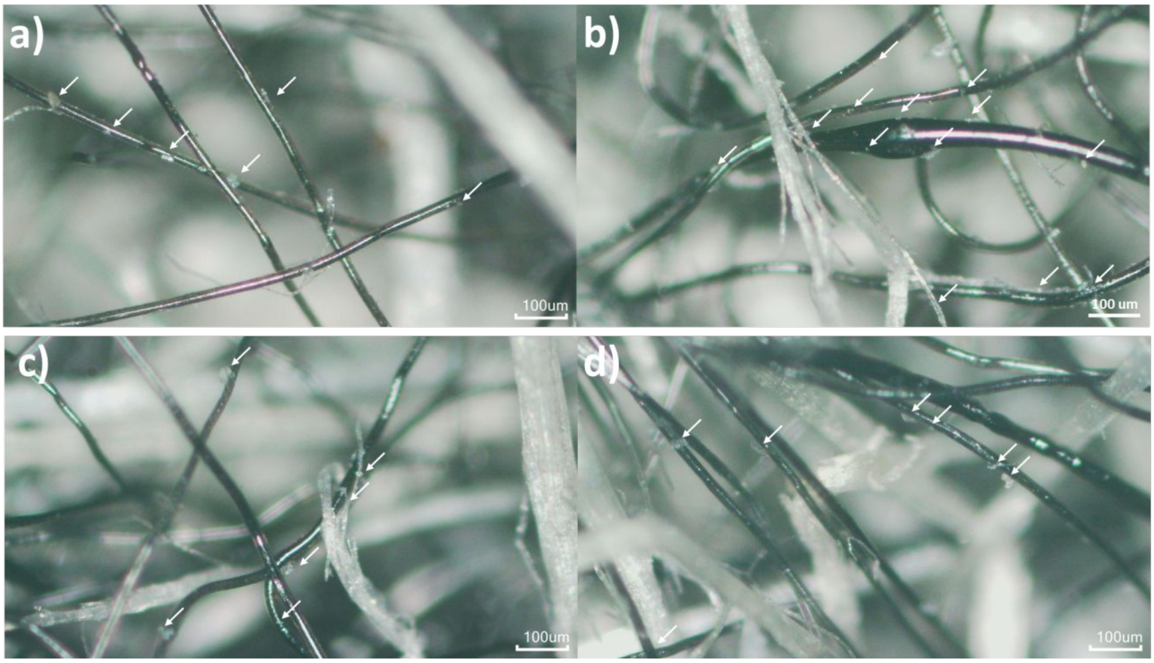

Figure S12. The OM images of 80PF_E_20LM_Oil_FR100_NW a-b) before and c-d) after washing.

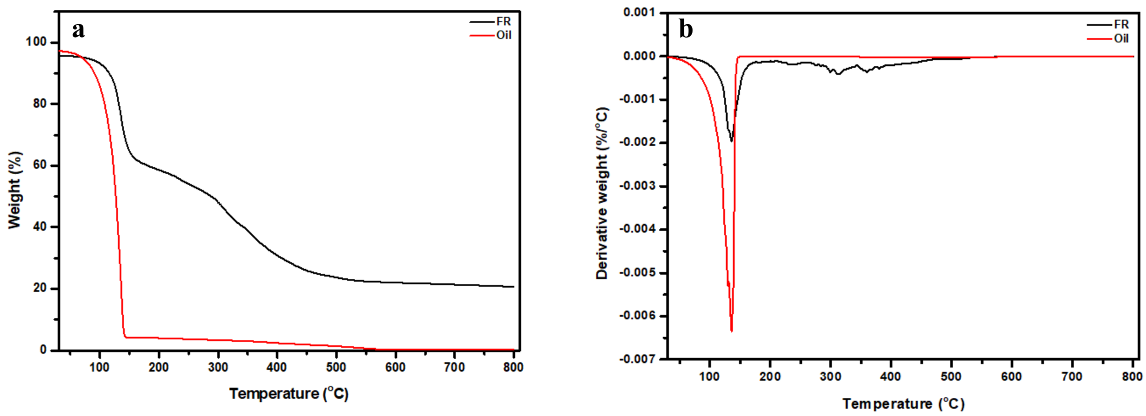

Figure S13. a) TGA and b) DTG of FR and Oil.

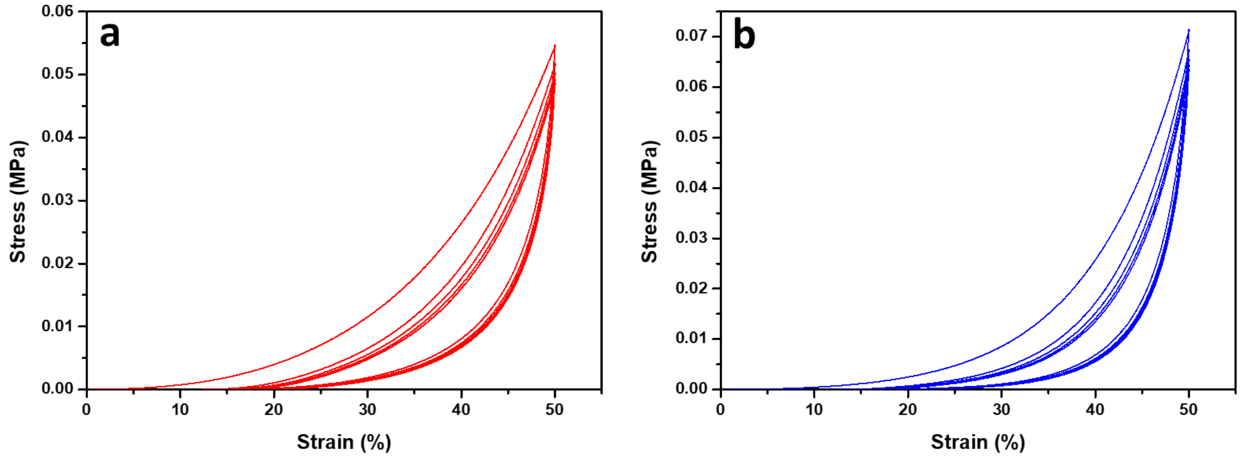

Figure S14. Curves of stress-strain at 50% strain amplitude under 5 compression cycles, a) 80PF_E_20LM_NW and b) 80PF_E_20LM_Oil_FR100_NW.

Table S2. Stress at max and energy absorption of 80PF_E_20LM_NW and 80PF_E_20LM_Oil_FR100_NW.

Cycle	**80PF_E_20LM_NW**		**80PF_E_20LM_Oil_FR100_NW**	
	Stress at Max (MPa)	Absorption Energy (J)	Stress at Max (MPa)	Absorption Energy (J)
1	0.056 ± 0.006	1.11 ± 0.12	0.071 ± 0.012	1.13 ± 0.20
2	0.053 ± 0.006	0.87 ± 0.09	0.068 ± 0.012	0.88 ± 0.15
3	0.051 ± 0.005	0.81 ± 0.09	0.065 ± 0.011	0.81 ± 0.14
4	0.051 ± 0.005	0.77 ± 0.08	0.064 ± 0.011	0.78 ± 0.13
5	0.050 ± 0.005	0.75 ± 0.08	0.064 ± 0.011	0.76 ± 0.13

Table S3. The sound absorption characteristic of PF_Com, PF_E, PF_E_Oil_FR100, 80PF_E_20LM_NW, and 80PF_E_20LM_Oil_FR100_NW

Sample	Sample thickness (mm)		
	10	30	50
PF_Com	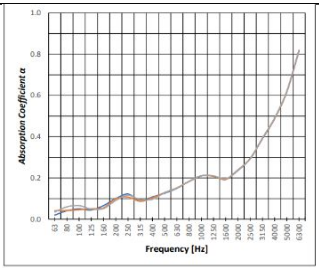	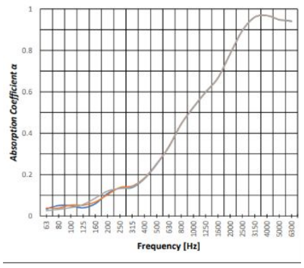	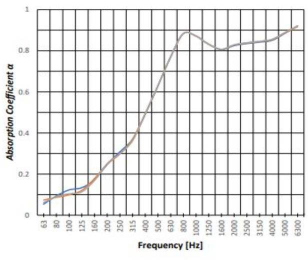
PF_E	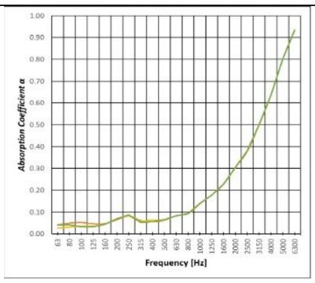	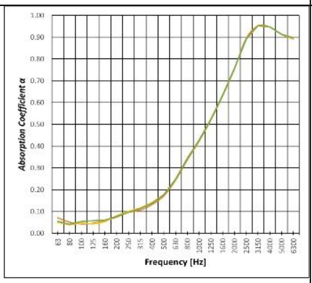	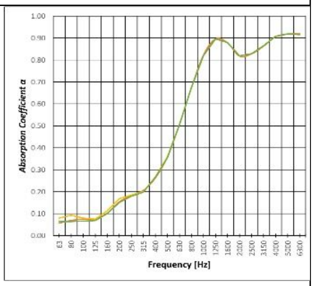
PF_E_Oil_ FR100	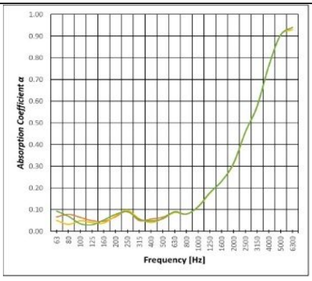	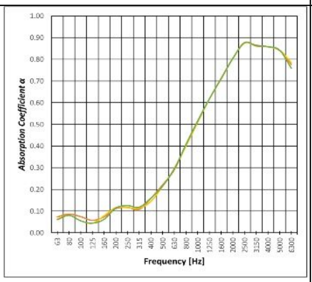	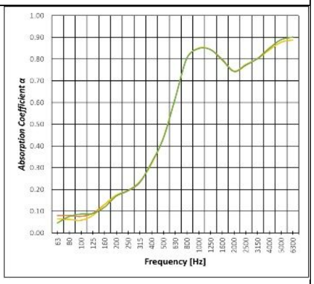
80PF_E_20LM_NW	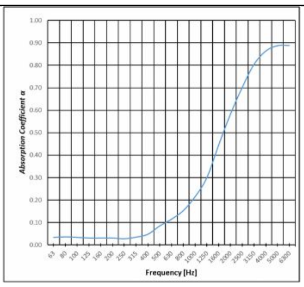	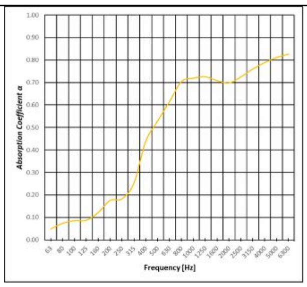	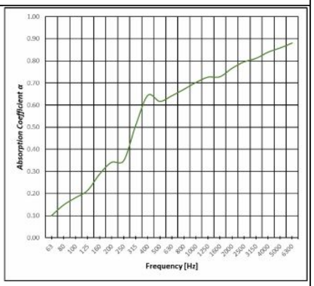
80PF_E_20LM_Oil_FR100_NW	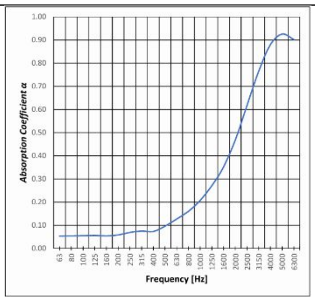	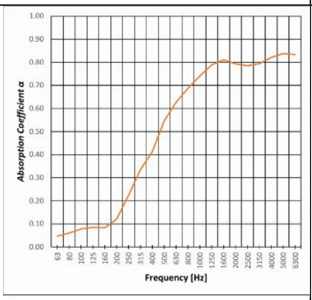	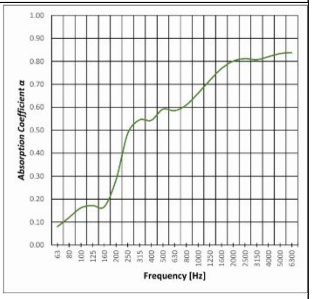
